# Safety and efficacy of BAY1436032 in IDH1-mutant AML: phase I study results

**DOI:** 10.1038/s41375-020-0996-5

**Published:** 2020-07-30

**Authors:** Michael Heuser, Neil Palmisiano, Ioannis Mantzaris, Alice Mims, Courtney DiNardo, Lewis R. Silverman, Eunice S. Wang, Walter Fiedler, Claudia Baldus, Sebastian Schwind, Timothy Pardee, Alexander E. Perl, Charles Cai, Stefan Kaulfuss, Eleni Lagkadinou, Christine Rentzsch, Markus Wagner, Gary Wilkinson, Bingyan Wu, Michael Jeffers, Isabelle Genvresse, Alwin Krämer

**Affiliations:** 1 Department of Hematology, Hemostasis, Oncology, and Stem Cell Transplantation, Hannover Medical School, Hannover, Germany; 2 Department of Medical Oncology, Sidney Kimmel Cancer Center, Thomas Jefferson University, Philadelphia, PA USA; 3 Department of Medical Oncology, Montefiore Medical Center, Albert Einstein College of Medicine, Bronx, NY USA; 4 Division of Hematology, Department of Medicine, The Ohio State University Comprehensive Cancer Center, Columbus, OH USA; 5 Department of Leukemia, The University of Texas M.D. Anderson Cancer Center, Houston, TX USA; 6 Tisch Cancer Institute, Division of Hematology/Oncology, Icahn School of Medicine at Mount Sinai, New York, NY USA; 7 Department of Medicine, Roswell Park Comprehensive Cancer Center, Buffalo, NY USA; 8 Department of Hematology and Oncology, University Hospital Hamburg-Eppendorf, Hamburg, Germany; 9 Division of Hematology and Oncology, Charité University Hospital Berlin, Berlin, Germany; 10 Division of Hematology and Oncology, University Hospital Leipzig, Leipzig, Germany; 11 Section on Hematology and Oncology, Comprehensive Cancer Center of Wake Forest Baptist Health, Winston-Salem, NC USA; 12 Division of Hematology-Oncology, Perelman School of Medicine, Abramson Cancer Center of the University of Pennsylvania, Philadelphia, PA USA; 13 Pharmaceuticals Division, Bayer HealthCare Pharmaceuticals, Inc., Whippany, NJ USA; 14 Pharmaceuticals Division, Bayer AG, Berlin, Germany; 15 Clinical Cooperation Unit Molecular Hematology/Oncology, German Cancer Research Center (DKFZ) and Department of Internal Medicine V, University of Heidelberg, Heidelberg, Germany

**Keywords:** Acute myeloid leukaemia, Drug development, Phase I trials

## Abstract

The mutant IDH1 (mIDH1) inhibitor BAY1436032 demonstrated robust activity in preclinical AML models, supporting clinical evaluation. In the current dose-escalation study, BAY1436032 was orally administered to 27 mIDH1 AML subjects across 4 doses ranging from 300 to 1500 mg twice-daily. BAY1436032 exhibited a relatively short half-life and apparent non-linear pharmacokinetics after continuous dosing. Most subjects experienced only partial target inhibition as indicated by plasma R-2HG levels. BAY1436032 was safe and a maximum tolerated dose was not identified. The median treatment duration for all subjects was 3.0 months (0.49–8.5). The overall response rate was 15% (4/27; 1 CRp, 1 PR, 2 MLFS), with responding subjects experiencing a median treatment duration of 6.0 months (3.9–8.5) and robust R-2HG decreases. Thirty percent (8/27) achieved SD, with a median treatment duration of 5.5 months (3.1–7.0). Degree of R-2HG inhibition and clinical benefit did not correlate with dose. Although BAY1436032 was safe and modestly effective as monotherapy, the low overall response rate and incomplete target inhibition achieved at even the highest dose tested do not support further clinical development of this investigational agent in AML.

## Introduction

Somatic hotspot mutations in isocitrate dehydrogenase 1 (*IDH1*) have been identified in a variety of cancers, with a frequency of ~7% in acute myeloid leukemia (AML) [1–6]. Tumor-associated *IDH1* mutations (*mIDH1*) change the conserved arginine at codon 132 in the enzymatic active site to a variety of alternative amino acids (R132X), and in doing so confer a neomorphic activity to this enzyme. Whereas wild-type IDH1 (wtIDH1) catalyzes the conversion of isocitrate to α-ketoglutarate (α-KG), mIDH1 converts α-KG to R-2-hydroxyglutarate (R-2HG). Subjects with *mIDH1* AML show elevated R-2HG levels, which inhibits α-KG-dependent enzymes, thereby leading to epigenetic alterations and ultimately impaired hematopoietic differentiation [7–14].

BAY1436032 is an oral small-molecule inhibitor of mIDH1 that is active in preclinical models of *mIDH1* cancer [15–17]. Most mIDH1 inhibitors, including BAY1436032, reportedly interact with an allosteric site on the mutant enzyme, although an inhibitor which interacts directly with the active site was recently described [18]. Preclinical experiments focusing on *mIDH1* AML found that BAY1436032 inhibits R-2HG production and colony growth *in vitro*, while promoting leukemic blast clearance, myeloid differentiation, and survival in animal models [16]. Supported by these encouraging preclinical findings, BAY1436032 was evaluated in a phase I clinical study in subjects with *mIDH1* AML (NCT03127735), the results of which are presented herein. Objectives of the study include determination of the maximum tolerated dose (MTD) and the recommended phase II dose (RP2D), and evaluation of the safety, tolerability, pharmacokinetics (PK), pharmacodynamics and clinical activity of BAY1436032.

## Materials and methods

### Study design

This study was an open-label, nonrandomized, multicenter phase I trial. Subjects were screened at 13 hospital sites in 2 countries (USA and Germany). The study was to consist of dose-escalation followed by dose-expansion. The MTD identified in dose-escalation was to be used in the dose-expansion. If MTD was not reached in dose-escalation, a dose for the expansion would be selected based on available PK, pharmacodynamic, safety, and efficacy data. BAY1436032 was administered twice-daily (BID) in continuous 28-day cycles. In dose-escalation, up to 9 evaluable subjects could be enrolled per cohort, with a minimum of 3 dose-limiting toxicity (DLT)-free evaluable subjects required prior to escalating to the next highest dose. Bayesian dose-DLT modeling was performed to help guide dosing decisions [19]. MTD was defined as the highest dose of BAY1436032 that could be given such that ≤25% of subjects were predicted to experience a DLT.

DLTs were differentially defined for nonhematopoietic versus hematopoietic toxicities. Nonhematopoietic toxicities of ≥grade 3 occurring during the first cycle of treatment were to be considered DLTs with the following exceptions: (1) Alopecia and nausea controlled by medical management; (2) Tumor lysis syndrome if successfully managed clinically and resolved within 7 days without any end-organ damage; (3) Differentiation syndrome (DS) if successfully managed clinically and resolved within 7 days without any end-organ damage; (4) Asymptomatic ≥grade 3 electrolyte abnormalities not considered clinically significant by the investigator. Missing >20% of doses of study drug due to any drug-related toxicity, or delay in the start of cycle 2 by more than 14 days due to any drug-related toxicity, were also considered DLTs. For certain toxicities such as laboratory assessments without a clear clinical correlate, a discussion between the investigator and the sponsor determined whether the adverse event (AE) should be assessed as a DLT.

For hematological toxicities, thrombocytopenia of ≥grade 3 with clinically significant bleeding, or grade 4 neutropenia persisting 42 days after the start of treatment in the absence of active AML, were considered DLTs.

DLTs identified during the first cycle of treatment were used to guide dose-escalation decisions and to determine the MTD, and if safety issues appeared in subsequent cycles they were also to be considered. Hydroxyurea was permitted during the first cycle if white blood cell (WBC) exceeded 20 × 10^9^/L and was also permitted for treatment of DS.

The study protocol was approved by the institutional review board of participating institutions and complied with the Declaration of Helsinki, current Good Clinical Practice guidelines, and local laws and regulations. Written informed consent was provided by all participants prior to the initiation of any study-specific procedure. Data were entered into clinical research forms by the study investigators and their staff. The study was sponsored by Bayer AG.

### Subjects

Male and female subjects of ≥18 years of age with an Eastern Cooperative Oncology Group (ECOG) performance status of ≤2 and advanced AML were eligible. Information regarding ELN 2010 risk classification was collected during screening [20]. Subjects were required to harbor a missense mutation in *IDH1-R132X* based on local testing reported by study investigators, with sponsor review of test results prior to enrollment. *IDH1* mutational status from a bone marrow sample collected during screening was retrospectively evaluated at Foundation Medicine. Subjects were to be relapsed or refractory to at least 1 previous line of therapy, or intolerable to or unable to receive established therapies, and could have received any number and type of prior therapies prior to enrollment, except those targeting mIDH1. A cohort sample size of 3 to 9 DLT-evaluable subjects in dose-escalation was chosen based on experience and simulation results from adaptive Bayesian dose-DLT model. This number of subjects is anticipated to provide sufficient safety information to help guide dose escalation decisions in a reasonable time frame without exposing an excess number of subjects to potentially toxic or inactive doses of study drug. Dose-expansion was not conducted in this study.

### Study assessments

The primary objectives of the study were to determine the safety, tolerability, MTD, and/or RP2D dose of BAY1436032 administered in a twice-daily dosing schedule in subjects with *mIDH1* advanced AML. Secondary objectives were to evaluate PK and to assess pharmacodynamic effects and evidence of clinical efficacy.

### Safety

Safety and tolerability were evaluated by analysis of adverse events, physical examinations, vital signs, ECOG performance status, and various laboratory assessments. For safety monitoring, subjects were scheduled for clinic visits every week for the initial three cycles of treatment, after which time visits could be reduced to every-other-week with investigator and sponsor agreement. Cardiac function was assessed with triplicate 12-lead electrocardiograms (ECG) at screening, C1-D1, C1-D2, C1-D15, D1 of every subsequent cycle, and at treatment end. Severity of adverse events and toxicities were graded by investigators according to the National Cancer Institute Common Terminology Criteria for Adverse Events (NCI-CTCAE) version 4.03. AEs are presented by the Medical Dictionary for Regulatory Activities (MedDRA) v21.1.

### Efficacy

Disease assessments and response evaluations from bone marrow aspirate or biopsy were scheduled at screening, C2-D1, C3-D1, D1 of every second cycle thereafter, and at treatment end (if not done on D1 of the last cycle). Peripheral blood was analyzed at each of these time points and at additional times between bone marrow assessments. Clinical efficacy was assessed by investigators using the modified 2003 International Working Group response criteria for AML [21] with some changes based on 2017 European Leukemia Net recommendations [22]. Response categories included: complete remission (CR), morphologic CR with incomplete hematological recovery (CRh), morphologic CR with incomplete platelet recovery (CRp), morphologic leukemia-free state (MLFS), partial remission (PR), stable disease (SD), and progressive disease (PD). Overall response rate included CR, CRh, CRp, MLFS, and PR. Following study completion, investigators provided survival information.

### Pharmacokinetics

Plasma samples were collected at various times on C1-D1, C1-D2, C1-D8, and C1-D15 and stored frozen for PK assessments. Collection times on these days are indicated on Supplementary Fig. S1 (C1-D8 PK results are not shown). The evening dose of BAY1436032 was withheld on C1-D1 to facilitate assessment of the 24-h single-dose time point on C1-D2. Quantitative analysis of BAY1436032 (free acid) in plasma was performed as described in Supplementary Methods.

### Pharmacodynamics

For quantification of R-2HG, plasma samples were collected at the following time points and stored frozen: screening, C1-D1 (pre-dose and post-dose), C1-D8 (pre-dose), C1-D15 (pre-dose and post-dose), C1-D22 (pre-dose), pre-dose on D1 and D15 of each cycle thereafter, and at treatment end. Plasma R-2HG concentrations were measured by Eurofins as described in Supplementary Methods.

### Retrospective mutational analysis

Mutational analysis was performed on bone marrow aspirates or biopsies collected during screening and on a subset of samples collected during BAY1436032 treatment. Testing was performed by Foundation Medicine using the FoundationOne Heme panel which detects alterations in >400 tumor-associated genes via next-generation sequencing. Information provided by Foundation Medicine included the allelic frequency and the likely pathogenic nature of each alteration identified.

## Results

### Subjects

**Fig. 1:**
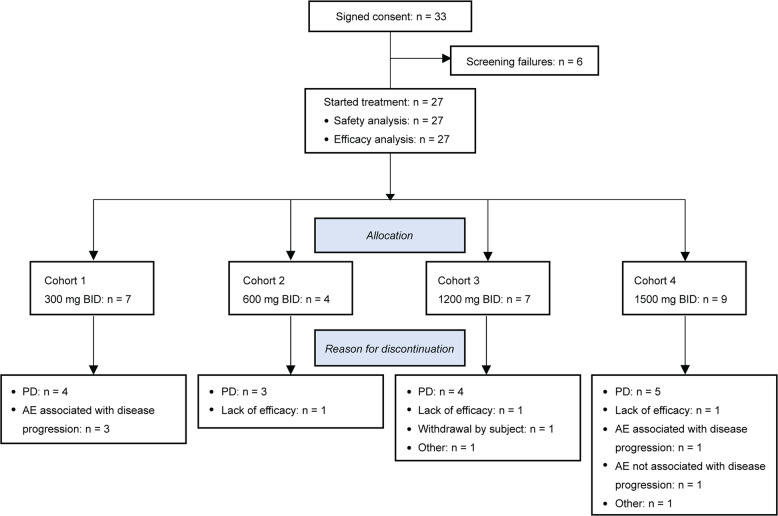
Subject disposition. The chart shows an overview of subjects who signed consent to participate in the study, their allocation into dose-escalation treatment cohorts, and reasons for their discontinuation from the study.

Thirty-three *mIDH1* AML subjects signed informed consent, 27 received BAY1436032 treatment and 6 failed screening for various reasons (e.g., presence of an uncontrolled infection). The first subject started treatment on June 28, 2017 and the last study visit was on December 5, 2018. All subjects were treated within dose-escalation, and a planned dose-expansion was not pursued. BAY1436032 tablets were orally administered BID, with the evening dose withheld on the first day of treatment to facilitate PK analysis. Administration was continuous and each treatment cycle was 28 days. Subjects were treated across 4 dosing cohorts: cohort 1 (300 mg BID; *n* = 7); cohort 2 (600 mg BID; *n* = 4); cohort 3 (1200 mg BID; *n* = 7) and cohort 4 (1500 mg BID; *n* = 9) (Fig. 1). The dosing schedule and starting dose were selected based on preclinical PK modeling and safety data, and on the results of the ongoing first-in-human phase I trial of BAY1436032 in subjects with *mIDH1* solid tumors (NCT02746081; [23]). Baseline demographics and disease characteristics are provided in Table 1. Subjects had received a median of 2 (0–8) prior systemic therapies for AML and 4 had received no prior systemic therapies. The prevalence of individual *IDH1-R132X* mutations across the 27 treated subjects based on investigator-reported information was as follows: R132C (*n* = 15), R132H (*n* = 5), R132G (*n* = 3), R132L and R132S (*n* = 2 each). Consistent with previous reports [4, 24], R132C and R132H were the most prevalent *IDH1* mutations identified.

**Table 1:** Baseline demographic and disease characteristics^a^.

	Cohort 1: 300 mg BID	Cohort 2: 600 mg BID	Cohort 3: 1200 mg BID	Cohort 4: 1500 mg BID	Total
	*n* = 7	*n* = 4	*n* = 7	*n* = 9	*n* = 27
Age [median (range), in years]	67 (37–86)	72 (51–76)	70 (42–83)	68 (27–79)	69 (27–86)
Sex, *n* (%)					
Male	4 (57)	2 (50)	3 (43)	3 (33)	12 (44)
Female	3 (43)	2 (50)	4 (57)	6 (67)	15 (56)
AML classification, *n* (%)					
De novo AML	5 (71)	3 (75)	4 (51)	7 (78)	19 (70)
Secondary AML	2 (29)	1 (25)	3 (43)	2 (22)	8 (30)
ECOG performance status, *n* (%)					
0	1 (14)	1 (25)	1 (14)	1 (11)	4 (15)
1	4 (57)	2 (50)	6 (86)	7 (78)	19 (70)
2	2 (29)	1 (25)	0	1 (11)	4 (15)
Time from initial diagnosis to 1st dose of study drug [median (range), in months]^b^	9 (1–32)	10 (2–19)	9 (1–25)	15 (5–44)	11 (1–44)
ELN risk classification, *n* (%)^c^					
Favorable	0	0	1 (14)	2 (22)	3 (11)
Intermediate	1 (14)	1 (25)	3 (43)	2 (22)	7 (26)
Adverse	4 (57)	2 (50)	2 (29)	5 (56)	13 (48)
Missing	2 (29)	1 (25)	1 (14)	0	4 (15)
mIDH1 identified, *n* (%)^d^					
R132C	3 (43)	2 (50)	5 (71)	5 (56)	15 (56)
R132H	3 (43)	0	0	2 (22)	5 (19)
R132G	1 (14)	2 (50)	0	0	3 (11)
R132L	0	0	2 (29)	0	2 (7)
R132S	0	0	0	2 (22)	2 (7)
Number of prior systemic antileukemic lines of therapies for AML, [median (range)]	1 (0–4)	1 (1–3)	2 (0–4)	3 (1–8)	2 (0–8)
Subjects having received at least 1 prior systemic antileukemic therapy for AML, *n* (%)	5 (71)	4 (100)	5 (71)	9 (100)	23 (85)
Prior allogeneic transplantation					
No	6 (86)	4 (100)	7 (100)	7 (78)	24 (89)
Yes	1 (14)	0	0	2 (22)	3 (11)

*BID* twice-daily, *ECOG* Eastern Cooperative Oncology Group, *ELN* European LeukemiaNet, *n* number of subjects.

^a^Percentages are calculated including missing values.

^b^For the calculation of time from initial diagnosis, only subjects with complete date information (year, month, day) are included.

^c^ELN classification as per 2010 recommendations [20].

^d^Investigator-reported *mIDH1* results used for subject enrollment are shown. Retrospective evaluation of baseline leukemic samples at a central laboratory via next-generation sequencing confirmed investigator-reported results in all evaluable cases.

### Pharmacokinetics and Pharmacodynamics

PK analysis was performed on C1-D1 after single-dose oral administration and on C1-D15 following continuous BID dosing. Following single oral administration, BAY1436032 plasma concentrations were detectable 30 min after administration. Maximum plasma concentrations were observed ~3 to 4 h after single-dose and continuous BID administration (Supplementary Fig. S1). In the evaluated dose range, BAY1436032 exposure after single-dose administration generally increased in a dose-proportional manner (1.6–2.0-fold); however, dose-proportionality was not apparent after continuous BID administration. Minimal accumulation was evident at C1-D15 and inter-subject variability was high in all cohorts for the main PK parameters evaluated (Supplementary Fig. S1 and Table S1).

To evaluate potential effects of the study drug on target inhibition, R-2HG levels were measured in plasma samples obtained at baseline and at various time points during treatment. Baseline R-2HG levels were highly variable, with a median concentration of 1755 ng/mL (78–14,749) (Supplementary Table S2). Of the 27 subjects treated, 26 had baseline R-2HG levels above those seen in *wtIDH1* cancers (61 ng/mL) and above the 97^th^ percentile upper reference limit found in healthy individuals (138 ng/mL) [25, 26].

All subjects achieved a lowering of baseline R-2HG levels during BAY1436032 treatment, with a median maximal decrease across all subjects of 66% (16–99) (Supplementary Table S2). However, only 5/26 subjects with an elevated baseline R-2HG level experienced a reduction to a normal level of ≤138 ng/mL. Maximal R-2HG decreases did not show a clear relationship with BAY1436032 dose, plasma exposure, or the specific *IDH1-R132X* mutation detected at baseline.

### Safety

Seventeen of 27 treated subjects experienced at least 1 treatment-emergent adverse event (TEAE) of any grade related to BAY1436032, with events occurring in ≥5% of treated subjects as listed in Table 2. Seven of 27 subjects experienced at least 1 TEAE of ≥grade 3 related to BAY1436032, with each of the following events occurring in a single subject: amylase increase, DS, fatigue, febrile neutropenia, hyponatremia, lung infiltration, peripheral edema, pneumonitis, and decreased WBC count (grade 3); anemia, ileus, decreased neutrophil count, decreased platelet count, and sepsis (grade 4). Twenty-four subjects experienced at least 1 TEAE ≥ grade 3 irrespective of relationship to study drug (Supplementary Table S3).

**Table 2:** BAY1436032-related treatment-emergent adverse events occurring in ≥5% of treated subjects.

TEAE	CTCAE grade	Cohort 1: 300 mg BID	Cohort 2: 600 mg BID	Cohort 3: 1200 mg BID	Cohort 4: 1500 mg BID	Total
		*n* = 7	*n* = 4	*n* = 7	*n* = 9	*n* = 27 (%)
Sinus tachycardia	Grade 1	1	0	0	1	2 (7)
Diarrhea	Grade 2	0	0	2	0	2 (7)
Nausea	Grade 1	1	1	1	1	4 (15)
	Grade 2	1	0	0	0	1 (4)
Vomiting	Grade 1	2	1	0	1	4 (15)
Fatigue	Grade 1	0	1	0	0	1 (4)
	Grade 2	1	0	1	0	2 (7)
	Grade 3	0	0	0	1	1 (4)
Decreased appetite	Grade 1	1	0	0	0	1 (4)
	Grade 2	1	0	0	0	1 (4)
Hypomagnesaemia	Grade 1	1	0	0	1	2 (7)
Differentiation syndrome^a^	Grade 2	0	0	0	1	1 (4)
	Grade 3	0	0	1	0	1 (4)
Dyspnea	Grade 1	1	0	1	0	2 (7)
	Grade 2	1	0	0	0	1 (4)

*BID* twice-daily, *CTCAE* common terminology criteria for adverse events, *n* number of subjects, *TEAE* treatment-emergent adverse events (as per MedDRA PT).

^a^Symptoms were not reported, only the term differentiation syndrome (DS). Three additional subjects presented with symptoms associated with DS and are not listed in the table since the individual symptoms occurred in <5% of treated subjects.

DS was considered an AE of special interest (AESI). Five of 27 (19%) treated subjects (2 each in cohorts 1 and 4, 1 in cohort 3) presented with DS, or symptoms associated with DS, which occurred between 8 and 76 days after the start of treatment. For treatment of DS symptoms, 4 subjects received steroids, 2 received hydroxyurea, and 1 had an interruption of study drug. All cases eventually resolved and 2 of the subjects experienced a clinical response (1 CRp, 1 MLFS).

No MTD was identified. A single DLT was reported: grade 4 ileus in subject 21 (cohort 4) who had a prior history of intestinal pseudo-obstruction. The ileus, which started on the 4th day of treatment, resolved following dose interruption and returned upon reintroduction of study drug. There were no clinically significant ECG such as prolongation of corrected QT interval related to BAY1436032 treatment.

Six of 27 subjects died either during BAY1436032 treatment or within 30 days of permanent treatment discontinuation, and none of these deaths were attributed to study drug. Causes of death were reported as PD or AE, in 3 subjects each. AEs associated with these deaths included sepsis, lung infection, and general physical health deterioration.

### Efficacy

The median treatment duration for all 27 subjects was 3.0 months (0.49–8.5) (Supplementary Table S2), and there was a trend towards increased neutrophil counts occurring during treatment (Supplementary Fig. S2).

**Fig. 2:**
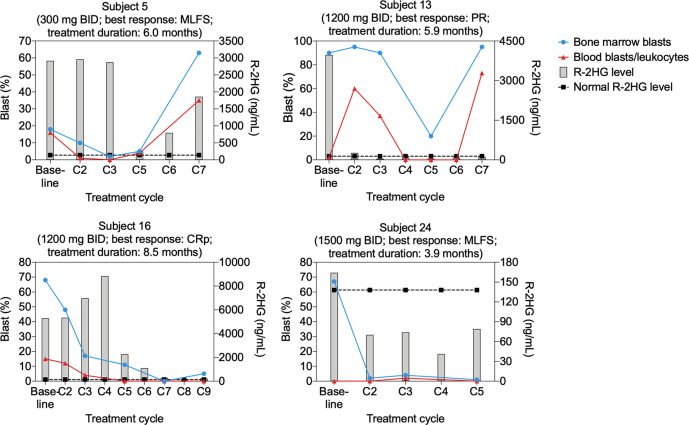
Per-subject blast percentages and R-2HG levels from subjects who experienced clinical responses during BAY1436032 treatment. Data from the four subjects who experienced clinical responses (1 CRp, 2 MLFS, 1 PR) is shown. Blast percentages are shown on the left *Y*-axis, plasma R-2HG levels on the right Y-axis and treatment cycle number on the X-axis. Bone marrow blasts are illustrated with blue circles and peripheral blood blasts/leukocytes with red triangles. R-2HG levels are represented by gray bars and the R-2HG level associated with healthy individuals (138 ng/mL) is shown as black squares connected by a dotted black line.

The overall response rate to BAY1436032, based on investigator assessment, was 15% (4/27): subject 16 (1200 mg BID) attained CRp at C7; subject 5 (300 mg BID) attained MLFS at C3; subject 24 (1500 mg BID) attained MLFS at C2 and went on to receive a 2nd stem cell transplant; and subject 13 (1200 mg BID) attained PR at C5 (Supplementary Table S2). The median treatment duration of these four responding subjects was 6.0 months (3.9–8.5). Blast counts and plasma R-2HG levels determined at various times during the treatment of these subjects are depicted in Fig. 2. In subject 13, R-2HG levels rapidly decreased to a normal level found in healthy individuals, whereas the reduction of R-2HG to a normal level took a longer time in subjects 5 and 16.

Best response of SD and PD was achieved by 18/27 and 5/27 subjects, respectively (Supplementary Table S2). Of the 18 subjects with SD, 8 remained stable for ≥2 consecutive monthly assessments, stayed on treatment for ≥3 months and are considered to have achieved relevant SD. This group of 8 subjects showed a median treatment duration of 5.5 months (3.1–7.0). Some decrease in leukemic blast counts and plasma R-2HG levels was evident in most of these subjects, but R-2HG levels generally did not reach normal levels (Supplementary Table S2, Figs. S3 and S4).

**Fig. 3:**
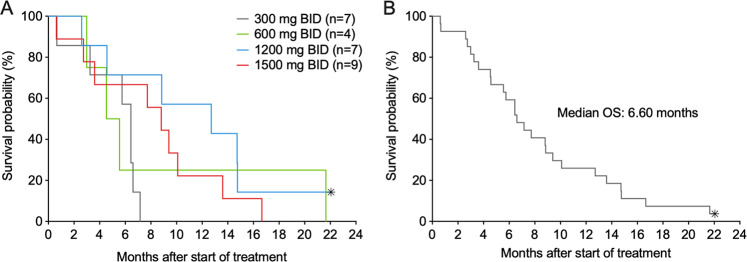
Subject survival after start of BAY1436032 treatment. Overall survival is depicted according to dosing cohort (**a**) and for all 27 treated subjects (**b**). Censored subjects are indicated with an asterisk.

As of November 20, 2019, 26/27 subjects were reported as deceased. Survival data by dosing cohort, as well as for the 27 subjects as a single group, is presented in Fig. 3. Median overall survival for the group of 27 treated subjects was 6.6 months (95% CI 4.6–9.4). The small subject numbers in each dosing cohort (*n* = 4–9) preclude relevant comparisons among cohorts.

### Retrospective mutational analysis

Following enrollment, retrospective mutational analysis of >400 tumor-associated genes was successfully performed on baseline leukemic samples obtained from 25/27 treated subjects and the *mIDH1* results from this analysis were concordant with those obtained locally via investigator assessment (Supplementary Tables S2 and S4).

Baseline leukemic samples from 24/25 of the subjects for whom retrospective mutational analysis was successfully performed harbored alterations in additional genes classified as known or likely pathogenic, with a median of 2.5 (0–4) co-occurring alterations detected per subject. Co-occurring alterations in the following genes were found in >10% of evaluated subjects: *DNMT3A* (*n* = 11), *ASXL1* (*n* = 7), *NRAS* (*n* = 5), *NPM1* (*n* = 5), *FLT3* (*n* = 4; 2 with an internal tandem duplication (ITD) and 2 with a kinase domain point mutation), *TET2* and *U2AF1* (*n* = 3 each). Co-occurring mutations in tyrosine kinase pathway genes such as *NRAS*, *FLT3*, and *PTPN11* were absent in responding subjects.

A co-occurring *IDH2-R140Q* mutation was detected in subjects 16 and 25, with variant allele frequencies (VAF) of 13% for *mIDH1*/26% for *mIDH2* in subject 16 and 5% for *mIDH1*/20% for *mIDH2* in subject 25. Subject 16 experienced a significant reduction in R-2HG levels during BAY1436032 treatment, achieved a clinical response (CRp) and remained on treatment for 8.5 months (Supplementary Table S2, Fig. 2). Subject 25, who had received the mIDH2 inhibitor enasidenib for the 10 months just prior to enrollment, experienced a modest reduction in R-2HG levels during BAY1436032 treatment, achieved a best response of SD and remained on treatment for 6.2 months (Supplementary Table S2, Fig. S4).

An analysis of baseline samples showed the median VAF of *mIDH1* to be 28% (5–49) (Supplementary Tables S2 and S4). The VAF of *mIDH1* in comparison to the VAF of co-occurring mutations suggested that *mIDH1* was present in the founding clone in 18/25 evaluated subjects. There was no apparent correlation between the *mIDH1* VAF and the specific *IDH1-R132X* mutation identified, or clinical outcome. Maximal R-2HG decreases detected during BAY1436032 treatment also did not correlate with baseline *mIDH1* VAF.

In addition to the analysis on baseline samples, for a limited number of subjects a retrospective mutational analysis was also performed on bone marrow samples collected during BAY1436032 treatment. Results from three subjects who had been on treatment for at least three full cycles of treatment and had data available from baseline, during treatment and end-of-treatment (EOT) are shown in Supplementary Fig. S5. Although the *IDH1* mutation present at baseline was always detected in samples collected during treatment and at the EOT, changes in other genes were evident: for subject 12 the EOT sample showed mutations in *WT1, BCOR*, and *FLT3* (ITD) and a *PDGFRB-TPM3* gene fusion that were undetectable at baseline or at C3-D1; for subject 14 the EOT sample showed a *MLL* duplication/rearrangement that was not detected at baseline or C3-D1; and for subject 18 a mutation in *STAG2* that was detected at baseline and at C3-D1 was not detected in the EOT sample, whereas the converse was true for mutations in *PIK3C2G* and *CREBBP*. Potential resistance mechanisms such as isoform switching and the acquisition of secondary alterations in *mIDH1* were evaluated and not detected.

## Discussion

In this study, the mIDH1 inhibitor BAY1436032 was evaluated at 4 different dose levels in a total of 27 AML subjects who harbored a mutation which altered the residue at position R132 of IDH1 to any one of 5 different amino acids, each of which is known to generate the R-2HG oncometabolite and to be inhibited by BAY1436032 [16, 17].

BAY1436032 was found to be generally safe, with just a single DLT (ileus) identified among the 27 treated subjects. The only other gastrointestinal-related TEAEs ≥grade 3 were two episodes of grade 3 nausea in cohort 4, which were not attributed to study drug. Each ≥grade 3 TEAE attributed to BAY1436032 occurred in a single subject. The most frequent ≥grade 3 TEAEs associated with ivosidenib, which is approved for a subset of subjects with *mIDH1* AML [27, 28], are QT prolongation (8%) and IDH DS (4%) [29]. Five subjects treated with BAY1436032 presented with DS, or symptoms associated with DS (all ≤grade 3), and all cases eventually resolved. There were no episodes of ≥grade 3 QT prolongation with BAY1436032. The favorable safety profile of BAY1436032 is consistent with preclinical studies demonstrating that BAY1436032 is a highly specific inhibitor of mIDH1 which exhibits little activity against wtIDH1 or wtIDH2 [16, 17].

Modest clinical benefit from single-agent BAY1436032 was identified in this study as evidenced by an overall response rate of 15% and a median treatment duration of 6.0 months among responders. Eight additional subjects who achieved SD for ≥2 consecutive monthly assessments and stayed on treatment for ≥3 months showed a median treatment duration of 5.5 months. Most of these eight subjects experienced decreases in both bone marrow and peripheral blood blast counts during treatment, consistent with some degree of clinical benefit from BAY1436032. Clinical outcome did not exhibit an obvious relationship to BAY1436032 dose.

Although BAY1436032 demonstrated some degree of R-2HG inhibition in all subjects, only 5/26 subjects with an elevated baseline R-2HG level experienced a reduction to a normal level during BAY1436032 treatment. In support of a potential relationship between the degree of target inhibition and clinical benefit, the four subjects who achieved clinical responses showed a median maximal inhibition of baseline R-2HG levels of 95% (75–99), compared with 58% (16–93) among the 23 other subjects. A correlation between the degree of R-2HG suppression and clinical response was not found for ivosidenib [29], although in that study R-2HG levels were strongly suppressed in the majority of subjects regardless of clinical response, thereby making it difficult to directly address this question.

It is unknown why a subset of subjects treated with BAY1436032 exhibit robust R-2HG suppression coupled with clinical benefit, while others do not. A dose-response relationship was not apparent when the degree of maximal percent R-2HG inhibition or the extent of clinical benefit was compared between cohorts, and subjects who achieved clinical benefit did not exhibit plasma concentrations markedly higher than those who did not.

The overall response rate of 15% for BAY1436032 is lower than the 33–42% reported for other mIDH1 inhibitors in relapsed and refractory AML [29–32], and the median overall survival (6.6 months; 95% CI 4.6–9.4) is somewhat lower than that reported for ivosidenib in similar populations (8.8 months; 95% CI 6.7–10.2) [29]. It is likely that the incomplete target inhibition generated by BAY1436032 in many subjects in the current study is a contributing factor towards the observed low response rate. Although the half-life of BAY1436032 could not be accurately determined due to insufficient elimination phase data, its half-life appears to be much shorter than that of ivosidenib (72–138 h; [29]) and it is clear that maximal BAY1436032 plasma levels significantly decrease during the administration intervals associated with BID dosing. However, the degree to which PK properties contribute to the differential levels of target inhibition, as indicated by decreased R-2HG levels, and clinical response rates achieved by BAY1436032 versus ivosidenib, is unknown. Some of the oncogenic properties of mIDH1 are independent of R-2HG [33], and the relative abilities of BAY1436032 and ivosidenib to inhibit R-2HG-independent oncogenic effects are not known. The finding that the number of colony-forming cells in primary AML samples are increased by ivosidenib (MH, unpublished) and enasidenib [34] but decreased by BAY1436032 [16], indicates that there may be inherent differences in important biological effects mediated by various mIDH inhibitors.

Retrospective mutational testing of baseline leukemic samples showed *mIDH1* results to be concordant with those that were obtained locally and used for enrollment. In addition to *mIDH1*, most subjects harbored alterations in one or more other genes commonly associated with AML. The median of 2.5 co-occurring mutations per subject, the prevalence of co-occurring mutations in genes such as *DNMT3A*, *ASXL1*, *NRAS*, and *NPM1*, and the absence of co-occurring mutations in tyrosine kinase pathway genes such as *NRAS*, *FLT3*, and *PTPN11* in responding subjects, is consistent with findings reported for ivosidenib [29, 35]. Co-occurring JAK2 mutations were associated with response to ivosidenib [35] and in the present study 1 of the 2 subjects with co-occurring JAK2 mutations exhibited a PR and remained on treatment for 5.9 months and the other achieved SD and remained on treatment for 5.5 months. The results in the current study are also consistent with a previous report that uncovered specific patterns of co-occurring mutations associated with specific *IDH1* mutations [24].

Retrospective mutational testing of samples collected during BAY1436032 treatment did not show *mIDH1* clearance over time as was identified in a subset of responders to ivosidenib [29], or evidence for acquired resistance via isoform switching or the acquisition of secondary alterations in *mIDH1* that were reported for ivosidenib [35–37]. Since these resistance mechanisms reportedly occur at low frequency, the lack of detection in the current study is not surprising given the small number of subjects and low response rate. The identification of changes in the mutational profile in some subjects treated with BAY1436032 indicates that targeted therapies may induce changes in the leukemic clonal composition relatively quickly, and even in the absence of formal clinical responses. Changes in molecular profiles following relatively short periods of therapeutic intervention were previously described for FLT3 inhibitors in FLT3-mutant AML [38].

Two subjects harbored activating mutations in both *IDH1* and *IDH2* at study entry. One of these subjects experienced a best response of SD and remained on treatment for 6.2 months. The other subject experienced a clinical response of CRp and remained on treatment for 8.5 months. Thus, although preclinical data demonstrates that BAY1436042 exhibits little or no inhibitory activity towards mIDH2 [16, 17], subjects with co-occurring mutations in *IDH1* and *IDH2* nonetheless appeared to benefit from BAY1436032. In the ivosidenib study, 2 subjects with co-occurring mutations in *IDH1* and *IDH2* showed a best response of SD [29].

Initial plans for this clinical study were to determine the MTD or RP2D in dose-escalation and then further evaluate BAY1436032 in additional AML subjects in dose-expansion. However, the results obtained in dose-escalation did not support initiating the dose-expansion part of the study. In addition to AML, BAY1436032 is also being evaluated in a phase I study of solid tumors, where objective responses have been observed in a subset of subjects with *mIDH1* glioma [23].

## Supplementary information

Supplementary Information

Supplementary Table
