# Supplementary material for: Safety and efficacy of BAY1436032 in IDH1-mutant AML: phase I study results
Source: Leukemia. 2020 Jul 30;34(11):2903–13. doi: 10.1038/s41375-020-0996-5 (PMC7584476; doi:10.1038/s41375-020-0996-5)

Supplementary Information for:

Safety and efficacy of BAY1436032 in IDH1-mutant AML: Phase I study results

Michael Heuser, Neil Palmisiano, Ioannis Mantzaris, Alice Mims, Courtney DiNardo, Lewis R. Silverman, Eunice S. Wang, Walter Fiedler, Claudia Baldus, Sebastian Schwind, Timothy Pardee, Alexander E. Perl, Charles Cai, Stefan Kaulfuss, Eleni Lagkadinou, Christine Rentzsch, Markus Wagner, Gary Wilkinson, Bingyan Wu, Michael Jeffers, Isabelle Genvresse, Alwin Krämer

SUPPLEMENTARY METHODS

Pharmacokinetics: Determination of BAY1436032 in plasma

Quantitative analysis of BAY1436032 (free acid) in plasma was determined using a fully validated method after protein precipitation with acetonitrile/0.4 mM ammonium acetate (pH 3, 80:20, v/v) containing the internal standard, followed by separation employing high-pressure liquid chromatography-tandem mass spectrometric detection (LC-MS/MS). The method validation and sample analysis were performed in compliance with the pertinent guidelines on Bioanalytical Method Validation of FDA (2001) and EMA (2011). Samples were stored at or below -15°C and analyzed within 135 days of sampling. Testing indicated that the BAY1436032 was stable for this period of time. Maximum drug concentration (C_{max}), area under the concentration versus time curve from time 0 to 8 hours ($AUC_{(0\text{-}8)}$), and AUC from time 0 to 12 hours ($AUC_{(0\text{-}12)}$) were calculated using the model-independent (compartment-free) method. The dose proportionality in the 300 mg to 1500 mg dose range was evaluated by performing explorative analyses of variance (ANOVA) on the log-transformed values of C_{max} and $AUC_{(0\text{-}12)}$, calculated from single and continuous BID dosing PK profiles.

Pharmacodynamics: Determination of R-2HG in plasma

Plasma R-2HG concentrations were measured by Eurofins using a qualified LC-MS/MS method with a lower limit of quantification (LLOQ) of 25 ng/mL. For assay validation purposes, plasma samples from 9 subjects with *wtIDH1* and *wtIDH2* cholangiocarcinoma who were tested showed a median R-2HG level of 44 ng/mL (32–64) and samples from

9 subjects with *mIDH1* cholangiocarcinoma showed a median R-2HG level of 181 ng/mL (43–1016).

SUPPLEMENTARY TABLES

Supplementary Table S1. Main pharmacokinetic parameters in plasma following single and continuous BID administration of BAY1436032.

C1-D1 (single-dose)					
		**Cohort 1 300 mg BID n=7**	**Cohort 2 600 mg BID n=4**	**Cohort 3 1200 mg BID n=7**	**Cohort 4 1500 mg BID n=9**
C_{max}	µg/L	1996 (106)	3199 (46)	5308 (143)	9822 (52)
AUC(0-8)	µg·h/L	6597 (107)	12520 (29)	20663 (131)	42160 (46)
AUC(0-12)	µg·h/L	7435 (99)	15186 (29)	24837 (130)	50285 (51)

C1-D15 (continuous BID dosing)					
		**Cohort 1 300 mg BID**	**Cohort 2 600 mg BID**	**Cohort 3 1200 mg BID**	**Cohort 4 1500 mg BID**
$C_{max,md}$	µg/L	1878 (100), n=6	5053 (75), n=4	6047 (126), n=7	9464 (74), n=7
$AUC(0\text{-}8)_{md}$	µg·h/L	5925 (124), n=5	22279 (101), n=3	22913 (140), n=6	33951 (84), n=5
$AUC(0\text{-}12)_{md}$	µg·h/L	6751 (131), n=5	25366 (101), n=3	21829 (150), n=5	44548 (68), n=3

Numbers represent geometric mean (CV).

Abbreviations - AUC: area under the concentration-time curve from zero to infinity after a single dose; AUC(0-8):AUC from time 0–8 h after a single dose; AUC(0-12):AUC from time 0–12 h after a single dose; $AUC(0\text{-}8)_{md}$: AUC from time 0–8 h after continuous BID dosing; $AUC(0\text{-}12)_{md}$: AUC from time 0–12 h after continuous BID dosing; BID: twice daily; C_{max}: maximum observed drug concentration in plasma after a single dose; $C_{max,md}$: C_{max} after continuous BID dosing; CV: coefficient of variation; n: number of subjects

Supplementary Table S2. Per-subject summary of baseline *mIDH1* allele frequency, clinical outcome, and R-2HG levels at baseline and during BAY1436032 treatment.

				Clinical outcome [2]		R-2HG [3]		
**BAY1436032 dose**	**Subject ID**	**Age/sex**	***mIDH1* (% VAF) [1]**	**Best response**	**Time on treatment (months)**	**Baseline (ng/mL)**	**Best decrease (ng/mL)**	**Cycle-day of best decrease (% change from baseline)**
**Cohort 1 300 mg BID**	1	79/M	R132C (44)	**SD**	7.03	9714	4057	C6-D1 (58)
	2	67/M	R132H (49)	PD	0.62	4558	2196	C1-D8 (52)
	3	69/F	R132C (10)	**SD**	3.09	2813	1526	C3-D1 (46)
	4	37M	R132H (10)	SD	2.00	78	46	C1-D15 (41)
	5	86/F	R132G (36)	MLFS	5.98	2914	259	C5-D1 (91)
	6	66/M	R132C (48)	**SD**	6.41	14749	2653	C4-D15 (82)
	7	57/F	R132H (38)	SD	1.81	1081	910	C1-D15 (16)
**Cohort 2 600 mg BID**	8	74/M	R132G (23)	SD	1.81	837	529	C1-D8 (37)
	9	76/F	R132C (41)	**SD**	4.57	3765	364	C5-D1 (90)
	10	51/M	R132G (35)	PD	3.32	1981	491	C4-D1 (75)
	11	70/F	R132C (27)	SD	1.77	1777	605	C1-D8 (66)
**Cohort 3 1200 mg BID**	12	62/M	R132C (28)	**SD**	5.52	1755	1482	C3-D1 (16)
	13	73/F	R132L (37)	PR	5.91	3969	33	C4-D15 (99)
	14	70/M	R132C (46)	**SD**	5.52	4207	2471	C3-D1 (41)
	15	83/F	R132C (21)	SD	1.11	510	138	C1-D8 (73)
	16	69/F	R132C (13)	CRp	8.54	5280	76	C8-D1 (99)
	17	82/M	R132L (25)	SD	3.02	332	61	C1-D8 (82)
	18	42/F	R132C (26)	**SD**	3.42	995	420	C1-D8 (58)
**Cohort 4 1500 mg BID**	19	68/F	R132S	SD	2.07	850	291	C1-D15 (66)
	20	67/F	R132H (45)	PD	0.99	833	207	C1-D8 (75)
	21	27/F	R132C (48)	PD	0.49	2025	1362	C1-D8 (33)
	22	52/F	R132H (9)	PD	0.85	887	193	C1-D8 (78)
	23	69/M	R132C (46)	SD	2.37	3599	265	C1-D8 (93)
	24	58/M	R132C	MLFS	3.88	164	41	C4-D1 (75)
	25	78/M	R132C (5)	**SD**	6.24	577	320)	C1-D15 (45)
	26	70/F	R132C (5)	SD	2.10	1060	401	C1-D8 (62)
	27	79/F	R132S (6)	SD	2.30	800	375	C1-D15 (53)

[1] Results are from retrospective evaluation of baseline leukemic samples at a central laboratory via next-generation sequencing, except subjects 19 and 24 for whom investigator-reported results are shown.

[2] Bone marrow efficacy assessments are performed at the start of each 28-day cycle. Best response is per Cheson criteria. Subjects who achieved a best response of SD and remained stable for ≥2 consecutive assessments and on treatment for ≥3 months are indicated with bolded SD. Time on treatment is measured from first to last dose of BAY1436032.

[3] R-2HG levels detected at baseline and at the time point showing the maximum R-2HG decrease from baseline are indicated. Plasma R-2HG levels determined at the following time points were used for this analysis: pre-dose at baseline (C1-D1), C1-D8, C1-D15, D1 and D15 of subsequent cycles.

Abbreviations - CRp: complete remission with partial platelet recovery; MLFS: morphologic leukemia-free state; PD: progressive disease; PR: partial remission; SD: stable disease; VAF: variant allele frequency

Supplementary Table S3. Treatment-emergent adverse events occurring in ≥10% of treated subjects.

TEAE	CTCAE grade	Cohort 1 300 mg BID n=7	Cohort 2 600 mg BID n=4	Cohort 3 1200 mg BID n=7	Cohort 4 1500 mg BID n=9	Total n=27 (%)
**Anaemia**	Grade 3	0	1	3	0	4 (15)
	Grade 4	1	0	0	0	1 (4)
**Febrile neutropenia**	Grade 3	2	1	2	2	7 (26)
**Leukocytosis**	Grade 2	1	0	1	0	2 (7)
	Grade 3	1	0	0	1	2 (7)
**Thrombocytopenia**	Grade 4	0	0	1	2	3 (11)
**Sinus tachycardia**	Grade 1	2	0	0	1	3 (11)
**Abdominal pain upper**	Grade 1	0	0	0	2	2 (7)
	Grade 2	0	0	0	1	1 (4)
**Constipation**	Grade 1	0	0	1	3	4 (15)
	Grade 2	1	0	0	0	1 (4)
**Diarrhea**	Grade 1	4	0	1	2	7 (26)
	Grade 2	0	0	2	1	3 (11)
**Nausea**	Grade 1	1	1	2	1	5 (19)
	Grade 2	1	0	0	1	2 (7)
	Grade 3	0	0	0	2	2 (7)
**Vomiting**	Grade 1	3	1	0	3	7 (26)
**Fatigue**	Grade 1	2	1	0	1	4 (15)
	Grade 2	1	0	2	1	4 (15)
	Grade 3	1	0	0	2	3 (11)
**General physical health deterioration**	Grade 3	0	0	0	2	2 (7)
	Grade 5	0	0	0	1	1 (4)
**Oedema peripheral**	Grade 1	3	1	2	0	6 (22)
	Grade 3	1	0	0	1	2 (7)
**Pyrexia**	Grade 1	0	1	1	2	4 (15)
	Grade 2	0	1	0	0	1 (4)
	Grade 3	0	0	0	3	3 (11)
**Gingivitis**	Grade 1	2	1	0	0	3 (11)
**Lung infection**	Grade 3	1	1	1	0	3 (11)
	Grade 5	1	0	0	0	1 (4)
**Pneumonia**	Grade 1	0	0	0	1	1 (4)
	Grade 2	1	0	1	0	2 (7)
	Grade 3	1	0	1	1	3 (11)
**Pneumonia fungal**	Grade 2	0	0	0	1	1 (4)
	Grade 3	0	0	0	1	1 (4)
	Grade 4	0	0	0	1	1 (4)

TEAE	CTCAE grade	Cohort 1 300 mg BID n=7	Cohort 2 600 mg BID n=4	Cohort 3 1200 mg BID n=7	Cohort 4 1500 mg BID n=9	Total n=27 (%)
**Sepsis**	Grade 4	2	1	0	0	3 (11)
	Grade 5	1	0	0	1	2 (7)
**Contusion**	Grade 1	1	0	1	1	3 (11)
	Grade 2	0	0	0	1	1 (4)
**Fall**	Grade 1	0	1	1	2	4 (15)
**Weight decreased**	Grade 1	2	0	1	0	3 (11)
	Grade 2	0	1	2	0	3 (11)
	Grade 3	0	0	0	1	1 (4)
**Decreased appetite**	Grade 1	3	0	0	2	5 (19)
	Grade 2	1	0	1	1	3 (11)
	Grade 3	0	0	0	1	1 (4)
**Hyperkalaemia**	Grade 1	1	1	0	0	2 (7)
	Grade 4	0	1	0	0	1 (4)
**Hyperuricaemia**	Grade 1	0	1	2	1	4 (15)
	Grade 3	0	1	0	0	1 (4)
**Hypokalaemia**	Grade 1	2	0	1	0	3 (11)
	Grade 2	0	0	0	1	1 (4)
	Grade 3	1	0	0	1	2 (7)
**Hyponatraemia**	Grade 1	2	0	0	0	2 (7)
	Grade 3	0	0	0	1	1 (4)
**Hypophosphataemia**	Grade 1	0	0	0	1	1 (4)
	Grade 2	1	0	0	1	2 (7)
**Arthralgia**	Grade 1	0	0	0	1	1 (4)
	Grade 2	0	0	1	1	2 (7)
**Pain in extremity**	Grade 1	1	0	1	0	2 (7)
	Grade 2	0	0	0	1	1 (4)
**Headache**	Grade 1	1	0	0	0	1 (4)
	Grade 2	2	0	0	0	2 (7)
	Grade 3	0	0	0	1	1 (4)
**Anxiety**	Grade 1	1	0	1	0	2 (7)
	Grade 2	0	0	0	1	1 (4)
**Acute kidney injury**	Grade 1	1	0	0	0	1 (4)
	Grade 2	1	0	0	0	1 (4)
	Grade 3	0	1	0	0	1 (4)
**Haematuria**	Grade 1	1	0	1	0	2 (7)
	Grade 2	0	1	0	0	1 (4)
	Grade 3	0	0	0	1	1 (4)
**Cough**	Grade 1	2	1	0	0	3 (11)
	Grade 2	1	0	0	1	2 (7)

TEAE	CTCAE grade	Cohort 1 300 mg BID n=7	Cohort 2 600 mg BID n=4	Cohort 3 1200 mg BID n=7	Cohort 4 1500 mg BID n=9	Total n=27 (%)
**Dyspnoea**	Grade 1	2	0	1	1	4 (15)
	Grade 2	1	0	1	0	2 (7)
	Grade 3	2	0	0	1	3 (11)
**Epistaxis**	Grade 1	0	1	0	2	3 (11)
	Grade 2	0	0	2	0	2 (7)
	Grade 3	0	1	0	0	1 (4)
**Productive cough**	Grade 1	0	1	1	0	2 (7)
	Grade 2	0	0	0	1	1 (4)
**Dry skin**	Grade 1	2	1	1	1	5 (19)
**Rash**	Grade 1	0	0	1	0	1 (4)
	Grade 2	0	1	0	2	3 (11)
**Hypertension**	Grade 2	0	0	0	2	2 (7)
	Grade 3	0	0	1	0	1 (4)

Abbreviations – BID: twice-daily; CTCAE: common terminology criteria for adverse events; n: number of subjects; TEAE: treatment-emergent adverse event

Supplementary Table S4. Baseline gene alterations in all subjects as determined by next-generation sequencing.

(Please see separate Table in Excel format)

SUPPLEMENTARY FIGURES

Supplementary Figure S1. Geometric mean plasma concentration-time profiles of BAY1436032 at dose levels of 300 mg to 1500 mg. (A) Single administration on C1-D1; (B) Continuous BID dosing to C1-D15. Data is plotted on both linear and semi-logarithmic scales, with BAY1436032 concentration illustrated on the Y-axis and sampling time after drug administration on the X-axis. Cohorts are represented as follows: cohort 1 (300 mg BID) by black circles; cohort 2 (600 mg BID) by green squares; cohort 3 (1200 mg BID) by blue triangles; cohort 4 (1500 mg BID) by inverted red triangles. Error bars illustrate the geometric standard deviation. Values below LLOQ were substituted by 1/2 LLOQ for the calculation of statistics. Abbreviations - h: hour; LLOQ: lower limit of quantification; n: number of subjects.

Supplementary Figure S1

A

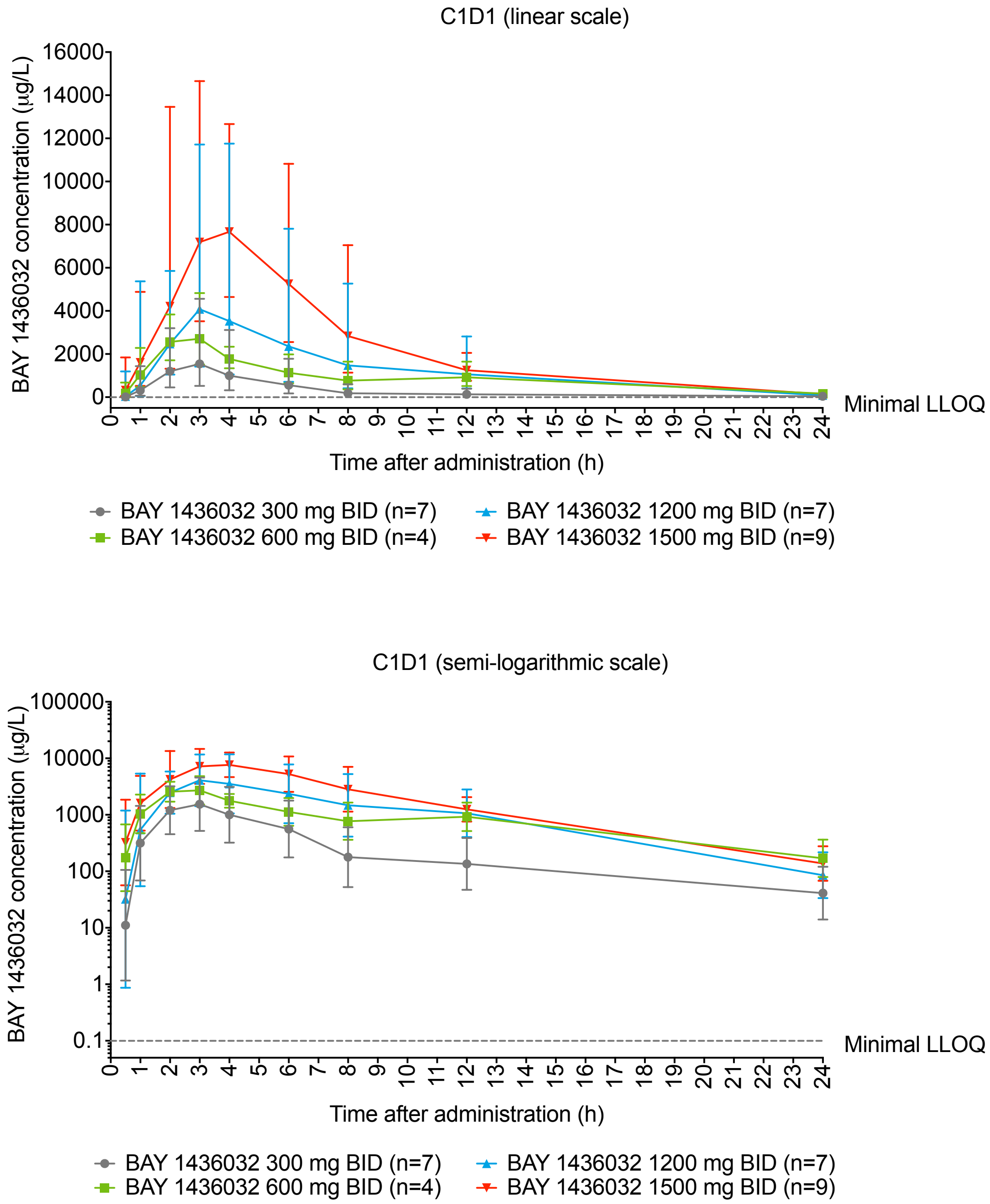

B

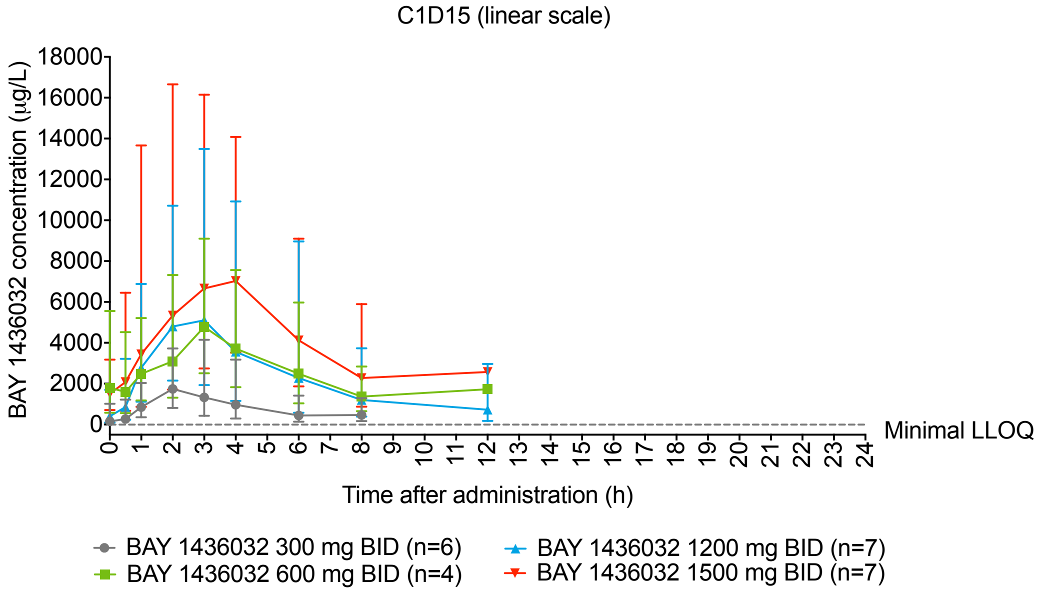
C1D15 (linear scale)
BAY 1436032 concentration (µg/L)
18000
16000
14000
12000
10000
8000
6000
4000
2000
0
Minimal LLOQ
0 1 2 3 4 5 6 7 8 9 10 11 12 13 14 15 16 17 18 19 20 21 22 23 24
Time after administration (h)
BAY 1436032 300 mg BID (n=6)
BAY 1436032 600 mg BID (n=4)
BAY 1436032 1200 mg BID (n=7)
BAY 1436032 1500 mg BID (n=7)

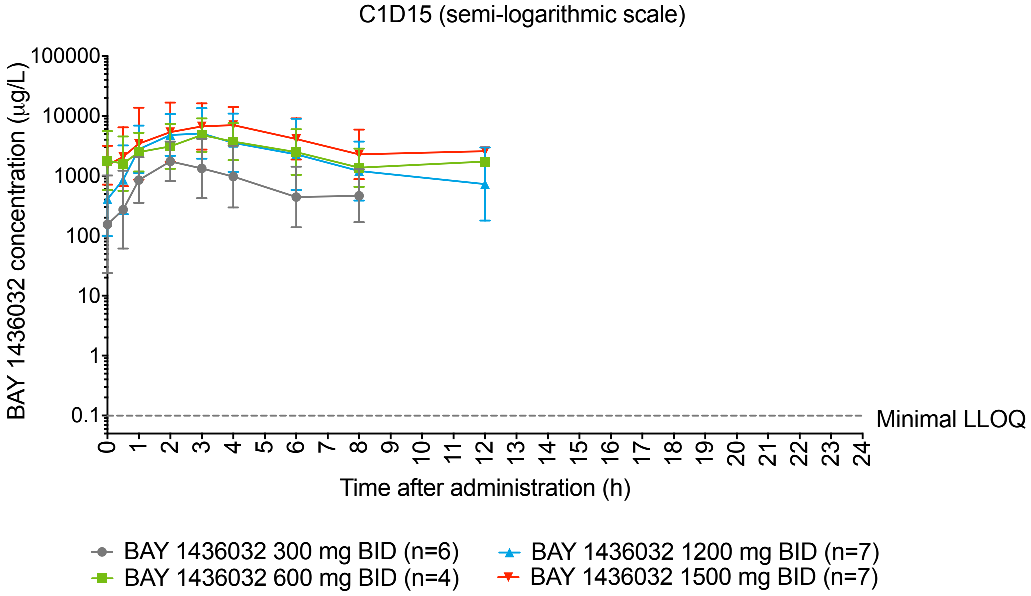
C1D15 (semi-logarithmic scale)
BAY 1436032 concentration (µg/L)
100000
10000
1000
100
10
1
0.1
Minimal LLOQ
0 1 2 3 4 5 6 7 8 9 10 11 12 13 14 15 16 17 18 19 20 21 22 23 24
Time after administration (h)
BAY 1436032 300 mg BID (n=6)
BAY 1436032 600 mg BID (n=4)
BAY 1436032 1200 mg BID (n=7)
BAY 1436032 1500 mg BID (n=7)

Supplementary Figure S2. Neutrophil counts during BAY1436032 treatment. All subjects with data available at each time point were included in the analysis. Neutrophils were quantified from blood samples obtained at the time points listed on the X-axis. Error bars indicate standard error of the mean. Abbreviations – n: number of subjects.

Supplementary Figure S2

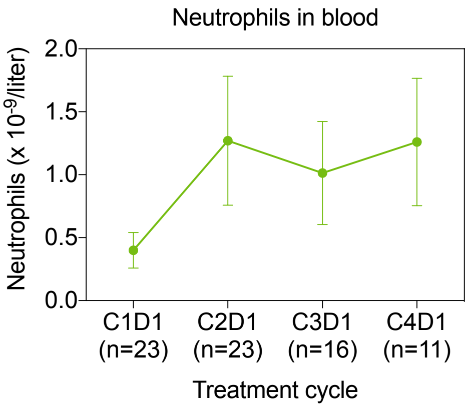

Supplementary Figure S3. Blast percentages in bone marrow and peripheral blood from subjects who experienced stable disease with BAY1436032 treatment. The 8 subjects who experienced a best response of SD and remained stable ≥2 consecutive assessments and on BAY1436032 treatment for ≥3 months were included in the analysis. Error bars indicate standard error.

Supplementary Figure S3

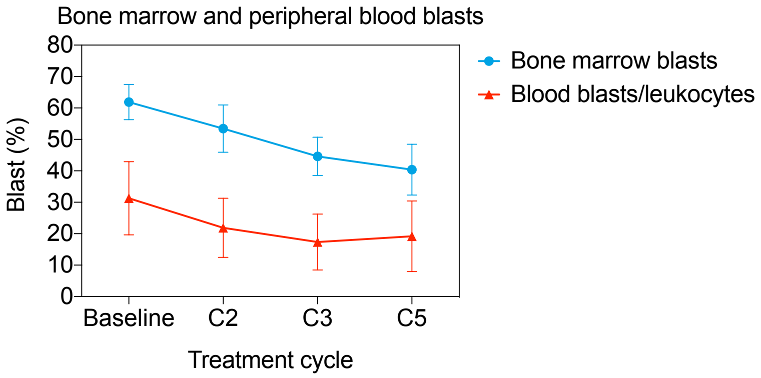

Supplementary Figure S4. Per-subject blast percentages and R-2HG levels from subjects who experienced stable disease during BAY1436032 treatment. The 8 subjects who experienced a best response of SD and remained stable ≥2 consecutive assessments and on BAY1436032 treatment for ≥3 months are depicted. Blast percentages are shown on the left Y-axis, plasma R-2HG levels on the right Y-axis and treatment cycle number on the X-axis. Bone marrow blasts are illustrated with blue circles and peripheral blood blasts/leukocytes with red triangles. R-2HG levels are represented by gray bars and the R-2HG level associated with healthy individuals (138 ng/mL) is shown as squares connected by a dotted black line. Note that some data points are missing (e.g. the R-2HG value for subject 12 at C5).

Supplementary Figure S4

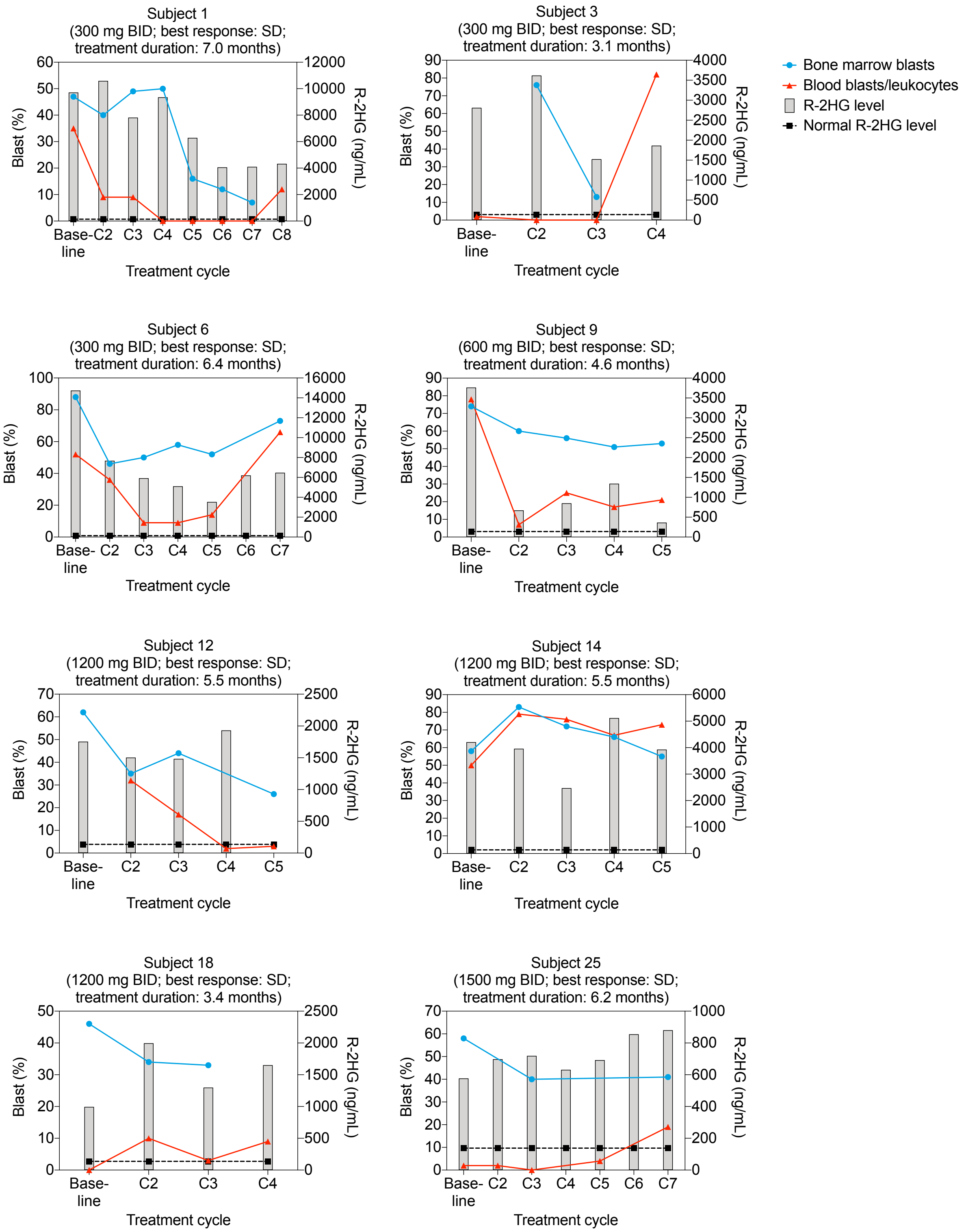

Supplementary Figure S5. Longitudinal mutational analysis during BAY1436032 treatment. Testing was performed on a subset of subjects and results are shown for the only 3 subjects who had been on treatment for at least 3 full cycles of treatment and had data available from multiple time points [baseline, during treatment, end-of-treatment, (EOT)]. The alterations listed are considered known or likely pathogenic, with the exceptions of those for *ATR* and *PIK3C2G* in subject 18. These alterations are included since they were previously identified as somatic in cancer specimens and the allele frequencies associated with these alterations in subject 18 are consistent with their being somatic alterations. Note that *PIK3C2G* and *CREB2* show overlapping variant allele frequency (VAF) values in subject 18. The *PDGFRB/TPM3* fusion and *MLL* rearrangement/duplication detected in subjects 12 and 14 only at EOT were not quantified for VAF and were assigned the VAF value of 50% in the graphs solely for illustrative purposes.

Supplementary Figure S5

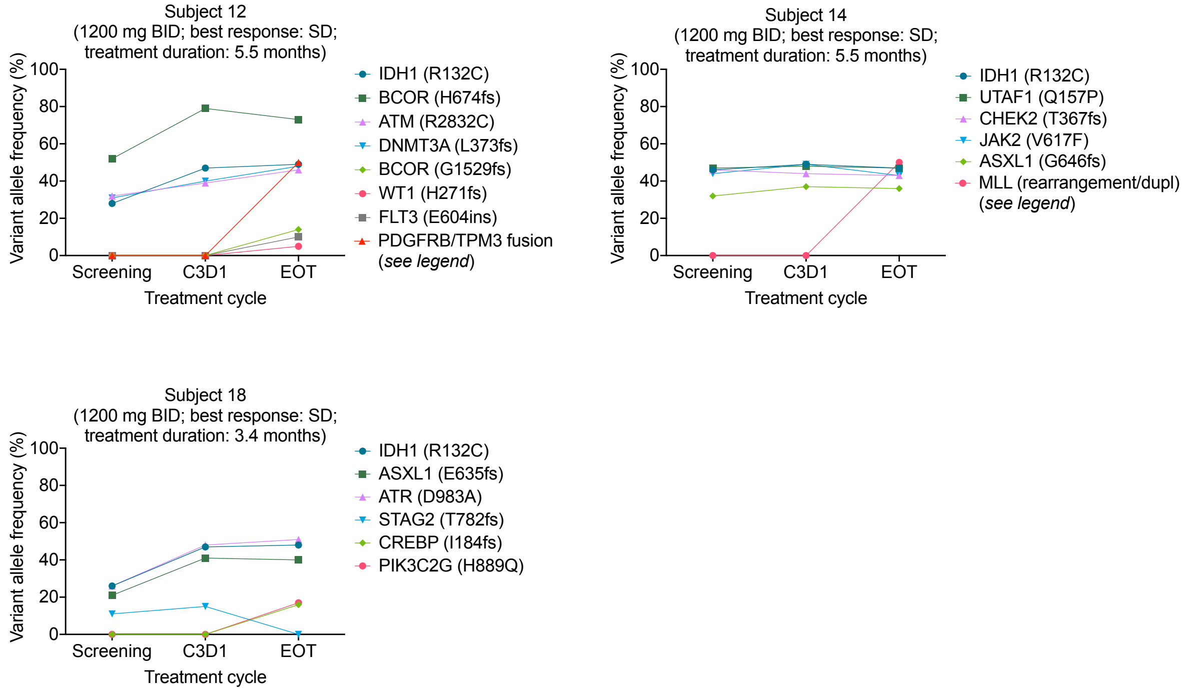

Supplement: Supplementary file 1 — Supplementary Information [file 41375_2020_996_MOESM1_ESM.pdf]
